# Effects of Different Brewing Technologies on Polyphenols and Aroma Components of Black Chokeberry Wine

**DOI:** 10.3390/foods12040868

**Published:** 2023-02-17

**Authors:** Mengying Chen, Shuting Zhang, Yuanxiao Ren, Zhao Le, Lingxi Li, Baoshan Sun

**Affiliations:** 1 School of Functional Food and Wine, Shenyang Pharmaceutical University, Shenyang 110016, China; 2 Pólo de Inovação de Dois Portos, Instituto Nacional de Investigação Agrária e Veterinária, I.P., Quinta da Almoinha, 2565-191 Dois Portos, Portugal

**Keywords:** black chokeberry, brewing technology, polyphenol, aroma

## Abstract

The black chokeberry is a shrub of the Rosaceae family, which is characterized by strong acidity and astringency and is widely processed into wine and alcoholic beverages. However, due to the characteristics of black chokeberries, the wine brewed by traditional methods often has a strong sour taste, weak aroma, and poor sensory quality. In order to improve the sensory quality and explore the effects of different brewing technologies on polyphenols of black chokeberry wine, five brewing technologies (traditional fermentation, frozen fruit fermentation, co-fermentation, carbonic maceration, and co-carbonic maceration) were used in this study. The results showed that compared with the traditional method, the four alternative brewing technologies could reduce acidity, increase the contents of several major polyphenols, and enrich floral scents and fruity aroma, thus significantly improving the sensory qualities of black chokeberry wine. The proposed brewing technologies would be applied to the production of quality black chokeberry or other fruit wines.

## 1. Introduction

The black chokeberry is a deciduous shrub belonging to the genus Sorbus of Rosaceae [1], originated in North America and introduced to China in the 1990s. Black chokeberry is rich in nutritive and bioactive substances, containing a variety of minerals, amino acids, and polyphenols [2,3]. Polyphenols in black chokeberry mainly include anthocyanins, proanthocyanidins and phenolic acids, with a total content of 1845–2340 mg GAE/100 g [4]. Polyphenols in black chokeberries have been reported to have beneficial effects on human health related to anti-inflammation, antioxidation, liver protection and urinary tract infection prevention [1,5]. However, due to the characteristics of strong acidity and astringency of black chokeberry fruits, they are usually not directly eaten, but processed into wine, jam, vinegar and other foods [6,7]. Compared with other types of fruit wine, black chokeberry wine may have higher nutritional value due to its richness in polyphenols. At present, black chokeberry wine is mainly brewed by the traditional fermentation method. The defects of high acidity and weak aroma of finished black chokeberry wine lead to its poor sensory quality, which is not widely accepted by the public. An alternative brewing technology, such as frozen fruit fermentation, co-fermentation, or carbonic maceration, may be a potential strategy to improve the sensory quality of black chokeberry wine.

Frozen fruit fermentation refers to the process of freezing the freshly harvested fruits for a period of time and then thawing them to use as brewing materials for alcohol fermentation. Freezing treatment can largely destroy the cytoarchitecture of berries, promote the release of aroma substances and polyphenols in cells, and improve the sensory quality of fruit wine [8]. Pedrosa-López M C et al. [9] reported that the aroma of the wine made from frozen berries was more prominent than that made from fresh berries.

Co-fermentation is a brewing method in which two or more kinds of brewing materials are mixed and macerated with a certain proportion and then alcoholic fermentation is started. This method can make use of the differences in the nutritional components of various winemaking materials and the advantages of their own flavors to cover up or make up for the shortcomings of single-fruit winemaking materials, improving the wine’s sensory quality [10,11].

Carbonic maceration is a red winemaking method invented by Michel Flanzy in the 1940s [12]. The intracellular fermentation method can effectively reduce the extraction of polyphenols in berries, significantly reduce the acidity and astringency of red wine [13], and make the wine soft and refreshing. In addition, the carbonic maceration process can give the wine a more fruity, floral fragrance [14], and enhance the aroma richness and complexity of the wine. Because the red wine produced by carbonic maceration has the characteristics of low acidity, strong aroma and soft taste [15], this method may have promising applications in the brewing of various fruit wines.

The objective of this work was to develop an alternative brewing technology to improve the sensory quality of black chokeberry wine. Five brewing methods including traditional fermentation (TF), frozen fruit fermentation (FF), co-fermentation (Co-F), carbonic maceration (CM) and co-carbonic maceration (Co-CM) were implemented and their effects on the phenolic composition and aroma profiles of black chokeberry wines were verified.

## 2. Materials and Methods

### 2.1. Black Chokeberries, Grapes and Chemicals

Cabernet Sauvignon grapes were harvested in September 2020 in a vineyard located in Qinhuangdao (Hebei, China). Black chokeberries were harvested in the same period in an orchard located in Haicheng (Anshan, Liaoning, China). The active dry yeast (commercial model number: RW) was purchased from Angel Yeast (Yichang, Hubei, China). Catechin was obtained from the Shanghai McLean Biochemical Technology Co. Ltd., Shanghai, China. Gallic acid was obtained from the Tianjin Guangfu Fine Chemical Research Institute. Chlorogenic acid, epicatechin, rutin hydrate, astragaloside, caffeic acid, quercetin, isoquercitrin and myricetin were obtained from the Chengdu Manchester Biotechnology Co. Ltd., Chengdu, China. The hydrocarbon mixture (C7-C40 alkanes) standard was obtained from the Shanghai Anpel Laboratory Technology Co. Ltd., Shanghai, China. Acetophenone, isopentyl acetate, benzyl alcohol, benzaldehyde, benzoic acid, 2,4-di-tert-butylphenol and 2-nonanone were obtained from the Beijing Tanmo Quality Inspection Technology Co. Ltd., Beijing, China.

### 2.2. Winemaking Processes

A total of five brewing methods including the traditional fermentation method (control), frozen fruit fermentation method, co-fermentation method, carbonic maceration method and co-carbonic maceration method were designed to explore the effect of these methods on the composition and content of polyphenols, aroma components and the sensory quality of black chokeberry wine.

#### 2.2.1. Traditional Fermentation

An amount of 30 kg of fresh black chokeberries were destemmed, crushed and placed in a 60 L stainless steel tank and treated with sulfur dioxide (50 mg/L). After 6 h, sugar was added into the tank to ensure that the total sugar content reached 230 g/L. After 6 h of maceration, dry yeast (240 mg/L) was added to start alcoholic fermentation at 25 °C. The cap was punched down three times daily until it remained submerged. When alcoholic fermentation was finished (residual sugar content <4 g/L), the mash was pressed. Free-run and press wines were combined and treated with sulfur dioxide (60 mg/L), then stored in vessels at 4 °C. After 6 months of maturation, the wine was racked and treated with sulfur dioxide (30 mg/L) and then bottled.

#### 2.2.2. Frozen Fruit Fermentation

Fresh black chokeberries were destemmed and placed in the −20 °C freezer. After two months, the frozen chokeberries were taken out to recover to room temperature. Then the 30 kg of berries were crushed and placed in a 60 L stainless steel tank and treated with sulfur dioxide (50 mg/L). After 6 h, sugar was added into the tank to ensure that the total sugar content reached 230 g/L. After 6 h of maceration, dry yeast (240 mg/L) was added to start alcoholic fermentation at 25 °C. The cap was punched down three times daily until it remained submerged. When alcoholic fermentation was finished (residual sugar content <4 g/L), the mash was pressed. Free-run and press wines were combined and treated with sulfur dioxide (60 mg/L), then stored in vessels at 4 °C. After 6 months of maturation, the wine was racked and treated with sulfur dioxide (30 mg/L) and then bottled.

#### 2.2.3. Co-Fermentation

An amount of 12 kg of fresh Cabernet Sauvignon grapes and 18 kg of fresh black chokeberry fruits were respectively destemmed, crushed and collected together in a 60 L stainless steel tank, and treated with sulfur dioxide (50 mg/L). After 6 h, sugar was added into the tank to ensure that the total sugar content reached 230 g/L. After 6 h of maceration, dry yeast (240 mg/L) was added to start alcoholic fermentation at 25 °C. The cap was punched down three times daily until it remained submerged. When alcoholic fermentation was finished (residual sugar content <4 g/L), the mash was pressed. Free-run and press wines were combined and treated with sulfur dioxide (60 mg/L), then stored in vessels at 4 °C. After 6 months of maturation, the wine was racked and treated with sulfur dioxide (30 mg/L) and then bottled.

#### 2.2.4. Carbonic Maceration

An amount of 5 kg of black chokeberries were crushed and collected in a 60 L stainless steel tank and treated with sulfur dioxide (50 mg/L). Then the remainder of the whole cluster of black chokeberries was carefully added into the same tank and stored at 25 °C under a CO_2_ atmosphere. After 3 days of intracellular fermentation/maceration, the mash was pressed. Free-run and press wines were combined, collected in the tank, and treated with dry yeast (240 mg/L) and sugar to undergo extracellular fermentation at 25 °C. Sugar was added into the tank to ensure that the total sugar content reached 230 g/L. When alcoholic fermentation was finished, the wine was treated with sulfur dioxide (60 mg/L) and stored in vessels at 4 °C. After 6 months of maturation, the wine was racked and treated with sulfur dioxide (30 mg/L) and then bottled.

#### 2.2.5. Co-Carbonic Maceration

An amount of 5 kg of Cabernet Sauvignon grapes were crushed and collected in a 60 L stainless steel tank and treated with sulfur dioxide (50 mg/L). Then, the remainder of the whole cluster of Cabernet Sauvignon grapes and the whole cluster of black chokeberries were carefully added into the same tank in a ratio of 3:2 and stored at 25 °C under a CO_2_ atmosphere. After 3 days of intracellular fermentation/maceration, the mash was pressed. Free-run and press wines were combined, collected in the tank, and treated with dry yeast (240 mg/L) and sugar to undergo extracellular fermentation at 25 °C. Sugar was added into the tank to ensure that the total sugar content reached 230 g/L. When alcoholic fermentation was finished, the wine was treated with sulfur dioxide (60 mg/L) and stored in vessels at 4 °C. After 6 months of maturation, the wine was racked and treated with sulfur dioxide (30 mg/L) and then bottled.

#### 2.2.6. Wine Sampling

All the following analyses on the wines were performed after two weeks of bottling. Moreover, the evolution of polyphenols during the alcoholic fermentation and maturation periods was also monitored.

### 2.3. Determination of Enological Parameters of Black Chokeberry Wines

The pH was determined by a digital pH meter (PB-10; Sartorius, Göttingen, Germany). Reducing sugar, total acid and alcohol in wine were measured according to OIV [16]. All determinations of wine indicators were repeated three times.

### 2.4. Determination of Total Polyphenols, Total Flavonoids, Total Proanthocyanidins and Total Anthocyanins of Black Chokeberry Wine

The total polyphenol contents in the wine were measured according to Gahler et al. [17]. The total flavonoid contents were measured according to Jia et al. [18]. The total proanthocyanidin contents were measured according to Sun et al. [19]. The total anthocyanin contents were determined by the pH difference method.

### 2.5. Analysis of Individual Phenolic Compounds

#### 2.5.1. Sample Pretreatment

The wine sample was dissolved in distilled water and lyophilized, after being evaporated to dryness under vacuum at 35 °C. Then, 10 mL of aqueous wine solution was loaded on a YMC ODS-A-HG (200 × 25 mm, 50 μm). Further, 200 mL of distilled water was added to remove the interfering components. Then 200 mL of ethyl acetate was used to elute the oligomeric polyphenol fraction, and 200 mL of methanol was used to elute the anthocyanin and polymeric polyphenol fraction. After solvent removal, distillate fractions were dissolved in distilled water and freeze-dried. The powders obtained were stored at −20 °C, ready for further analysis.

#### 2.5.2. Qualitative Analysis of Anthocyanins

UPLC Q-Exactive Orbitrap equipment (Waters, Milford, MA, USA) coupled with an electric spray ion source was used for the qualitative analysis of anthocyanins. The separation was performed on a UPLC-BEH C18 Column (2.1 mm × 50 mm, 1.7 μm) with the mobile phases of A (water: formic acid; 99.8: 0.2, *v*/*v*) and B (acetonitrile: water; 60:40, *v*/*v*). The gradient elution program: 0 min (A 82%: B 18%), 6 min (A 73%: B 27%), 12 min (A 65%: B 35%), 15 min (A 10%: B 90%), 22 min (A 0%: B 100%), with a flow rate of 0.3 mL/min and an injection volume of 3 μL. The column temperature was 30 °C. Detection wavelength was 525 nm. The parameter settings for H-ESI were as follows: detection in positive ion mode; capillary voltage: +3 kV, −2.5 kV; sheath gas: 45 psi; auxiliary gas: 15 arb; capillary temperature: 350 °C; H-ESI heating temperature: 300 °C. Quality scanning range: 100–1500 m/z, detection mode: Full MS/dd-MS2, Full MS resolution: 70,000, dd-MS2 resolution: 17,500.

#### 2.5.3. Quantitative Analysis of Phenolic Compounds

HPLC equipment (Waters e2695, Milford, MA, USA) was used for the quantitative analysis of anthocyanins and non-anthocyanic phenolic compounds. By liquid chromatography, the mixed solution of chlorogenic acid, epicatechin, rutin, astragalin, caffeic acid, quercetin, isoquercitrin and myricetin solution was used as the control solution of non-anthocyanins. The mixed solution of cyanidin-3-O-galactoside, cyanidin-3-O-glucoside and cyanidin-3-O-arabinoside solution was used as the reference solution of anthocyanins.

The separation was carried out using the mobile phases A (water: formic acid; 97:3, *v*/*v*) and B (acetonitrile: water; 60:40, *v*/*v*) on an Aglient Zorbax SB-C18 Column (4.6 × 250 mm, 5 μm). With a flow rate of 1 mL/min and an injection volume of 3 μL, the gradient elution program was as follows: 0 min (A 82%: B 18%), 6 min (A 77%: B 23%), 12 min (A 75%: B 25%), and 26 min (A 0%: B 100%). The temperature in the column was 30 °C. For the non-anthocyanins, the quantification was done at 525 nm for anthocyanins, and at 320 nm and 280 nm for phenolic acids and other polyphenols, respectively.

### 2.6. GC–MS Analysis of Aroma Components

#### 2.6.1. Sample Pretreatment

The wine samples were first pre-treated by liquid–liquid extraction. In brief, 10 mL of the wine sample was extracted three times with dichloromethane at a volume ratio of 1:1. The extract was combined, condensed to 2 mL, filtered, and then subjected to a GC–MS analysis.

#### 2.6.2. Qualitative Analysis of Aromatic Components

We prepared a 3% C7~C40 normal alkanes standard solution as the normal alkanes standard solution. The DB-Wax chromatographic column (30 m × 0.25 mm, 0.25 μm) was used with the GC–MS equipment (Thermo Trace 1300-ISQ; Thermo Technology Co., Ltd., Waltham, MA, USA). The oven’s temperature was set for an initial temperature of 45 °C for 2 min, increased at a rate of 2 °C /min to 160 °C for 3 min, then increased at a rate of 3 °C/min to 210 °C for 6 min, and finally maintained at 230 °C for 6 min. N_2_ (purity ≥ 99.996%) was used as the carrier gas with a flow rate of 1 mL/min and a 1:1 split ratio. The injection port had a temperature of 240 °C. Electronic impact ionization was used to carry out the mass spectrometry detection (70 eV). Injector, quadrupole, and ion source temperatures were, respectively, 240 °C, 150 °C, and 230 °C. The scanning range used to gather the data was 33–450 amu (atomic mass unit).

#### 2.6.3. Quantitative Analysis of Aromatic Components

The same GC–MS equipment and chromatographic column used in Section 2.6.2 were used for the quantitative analysis of aromatic components. The oven’s temperature was set to an initial temperature of 45 °C for 10 min, then increased at a rate of 2 °C/min to 230 °C for 10 min. Helium (purity ≥ 99.996%) was used as the carrier gas, with a flow rate of 1 mL/min and a 1:1 split ratio. The injection port had a temperature of 230 °C. Electronic impact ionization was used to carry out the mass spectrometry detection (70 eV). Injector, quadrupole, and ion source temperatures were, respectively, 230 °C, 150 °C, and 230 °C. The scanning range used to gather the data was 33–450 amu (atomic mass units). The identifications of volatile aroma compounds were based on the comparison of retention times and mass spectra with those of pure standards in the NIST2017 library. Quantification was performed according to the internal standard (Acetophenone) method, and the standard curve was plotted using the 5-point method. The results were expressed as mg/L of wines.

### 2.7. Sensory Evaluation by Tasting Panel

The tasting table (Table 1) for the sensory analysis of the aroma, flavor and color of the wine was established based on the Wine Tasting Table of American Wine Society (AWS) and with reference to the Wine Aroma Wheel (U.C. Davis Aroma Wheel). The sensory panel consisted of nine qualified tasters with certificates of the Wine & Spirit Education Trust (WSET) Award in Wines. Each member of the group tasting panel had participated in five 60 min professional training courses before the sensory evaluation test [20]. The quality of wine was rated by each panelist. The average score was used to represent each panelist’s quantifiable value. Table 1 contains scores on a 20-point scale.

### 2.8. Statistical Analysis

Each of the brewing technologies was carried out in duplicate. Sampling and analyses were performed in triplicate, and the data are presented as mean ± SD. Significant difference was compared by the Duncan test of one-way ANOVA (*p* < 0.05). SPSS 17.0 software was used for statistical analysis.

## 3. Results

### 3.1. Dynamic Monitoring of Polyphenols in Black Chokeberry Wines during Alcoholic Fermentation and Maturation Period

#### 3.1.1. Variation of Total Polyphenols, Total Proanthocyanidins, Total Anthocyanins and Total Flavonoids

**Figure 1:**
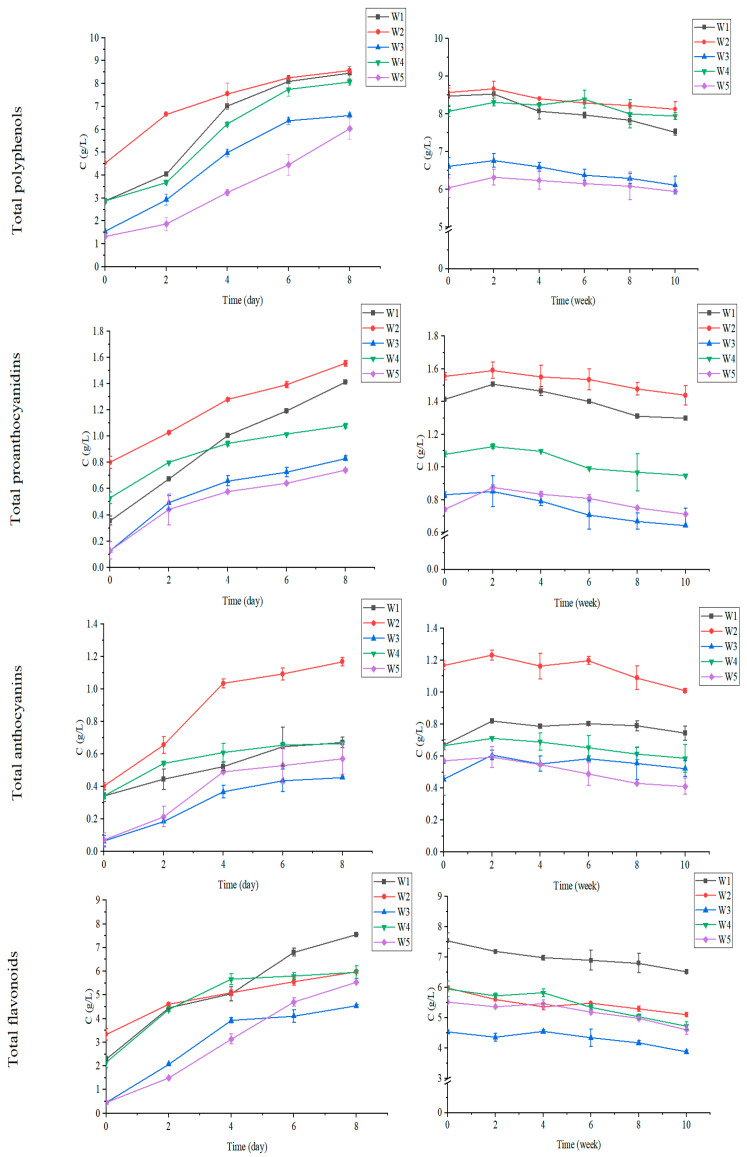
Total polyphenols, total proanthocyanidins, total anthocyanins and total flavonoids in black chokeberry wine. The left graph shows the alcoholic fermentation stage; the right graph shows the maturation stage. W1—wine made by traditional fermentation; W2—wine made by frozen fruit fermentation; W3—wine made by co-fermentation; W4—wine made by carbonic maceration; W5—wine made by co-carbonic maceration.

Variations of total polyphenols, total proanthocyanidins, total anthocyanins and total flavonoids from fermentation to maturation were monitored. Figure 1 shows that there was a significant change trend in the total phenolic content of black chokeberry wines during the alcoholic fermentation and maturation periods. The total polyphenols, total proanthocyanidins, total anthocyanins and total flavonoids of the five wines showed an overall trend of increasing during the alcoholic fermentation and then decreasing during the fermentation and maturation stages of the black chokeberry wines. This is because in the fermentation stage of black chokeberry wine, the alcoholic fermentation and maceration of solids promote the dissolution of phenolic components in berries; whereas in the maturation stage, the occurrence of oxidation, auxiliary color, and condensation reactions among phenolic components lead to the formation of precipitation of some phenolic substances, thus showing a tendency to rise and then fall.

As for total polyphenols, wine made by FF showed the highest total polyphenol content, followed by CM, TF, Co-F and Co-CM. The total polyphenol contents of wines made by FF and CM were higher by 8.1% and 5.7%, respectively, compared with that made by TF. The total anthocyanin content of the black chokeberry wine brewed by FF was the highest, and the content of total proanthocyanidins was increased by 11% compared with that brewed by TF. The total proanthocyanidins and anthocyanins in the wines made by the other three methods were lower than those made by traditional fermentation. As for total flavonoids, compared with the TF, the four alternative fermentation methods did not increase their contents. Among all the methods, FF was the most effective in increasing the total content of anthocyanins, polyphenols and proanthocyanidins in black chokeberry wine, whereas TF favored the retention of flavonoids, and CM tended to increase the total polyphenol content.

#### 3.1.2. Variation of Individual Phenolic Compounds

##### Variation of Anthocyanins

Anthocyanins are important components that give color to the wine of black chokeberries. Anthocyanins in nature are presented in the form of anthocyanidin glycosides or acylated anthocyanins. The most common types of anthocyanidins (known as aglycone) are namely cyanidin (CY), delphinidin (DP), pelargonidin (PG), malvidin (MV), peonidin (PN) and petunidin (PT).

In this study, various anthocyanins were identified by UPLC Q-Exactive Orbitrap in different wine samples. The results are presented in Table 2. Compounds 2, 3 and 4 were identified by comparison with the standard substances as cyanidin-3-*O*-galactoside, cyanidin-3-*O*-glucoside and cyanidin-3-*O*-arabinoside, respectively. Compounds 1 and 6 had the same MS/MS fragment ion of m/z 287, which corresponded to cyanidin (CY). Compound 1 with [M+H]^+^ ion at m/z 611.1611 caused a typical cleavage of glycosidic bond to produce [M-glucose]^+^ and [M-2×glucose]^+^, which was identified as cyanidin-3,5-*O*-diglucoside. The molecular ion obtained from compound 6 was m/z 419.0978, and its fragment ion at m/z 287.0550 was observed due to the loss of pentose moiety. Based on previously published data, it was inferred as cyanidin-3-*O*-xyloside [21]. Similarly, the compound 5 [M+H]^+^ ion at m/z 493.1357, compound 7 [M+H]^+^ ion at m/z 625.1713 and compound 8, [M+H]^+^ ion at m/z 655.1654 displayed identical fragmentation features, and, according to literature data, they were further confirmed as malvidin-3-*O*-glucoside, peonidin-3,5-*O*-diglucoside and malvidin-3-(6”-caffeoyl)-glucoside [22,23]. Cyanidin-3,5-*O*-diglucoside, cyanidin-3-*O*-galactoside, cyanidin-3-*O*-glucoside, cyanidin-3-*O*-arabinoside and cyanidin-3-*O*-xyloside were detected in five black chokeberry wines. Malvidin-3-*O*-glucoside, peonidin-3,5-*O*-diglucoside and malvidin-3-(6”-caffeoyl)-glucoside were only detected in wines made by Co-F and Co-CM. These three anthocyanins were lacking in black chokeberries but present in Cabernet Sauvignon grapes. Therefore, the above three anthocyanins were not detected in TF, FF and CM wines.

**Figure 2:**
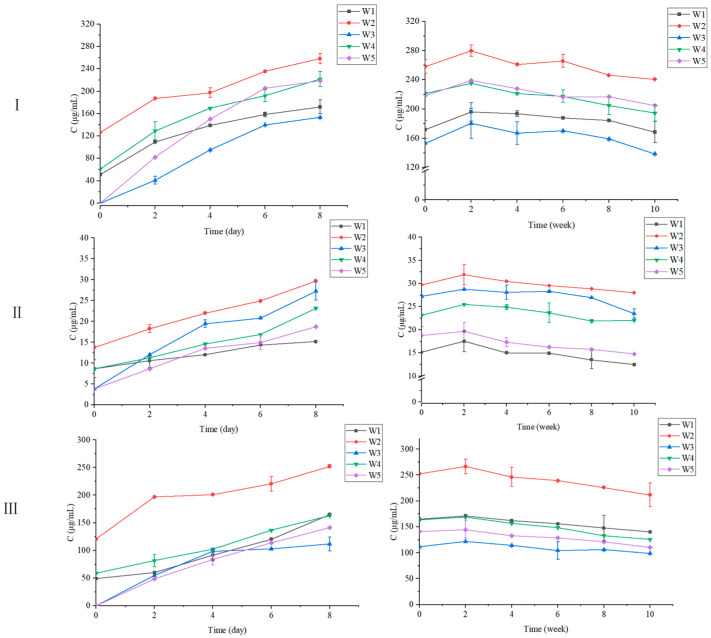
Variation in the contents of the major anthocyanins in black chokeberry wines during alcoholic fermentation period (left graph) and maturation period (right graph). (**Ⅰ**). Cyanidin-3-*O*-galactoside, (**Ⅱ**). Cyanidin-3-*O*-glucoside, (**Ⅲ**). Cyanidin-3-*O*-arabinoside. W1—wine made by traditional fermentation; W2—wine made by frozen fruit fermentation; W3—wine made by co-fermentation; W4—wine made by carbonic maceration; W5—wine made by co-carbonic maceration.

As shown in Figure 2, the results indicated that regarding the content of the cyanidin-3-*O*-galactoside, cyanidin-3-*O*-glucoside and cyanidin-3-*O*-arabinoside, the five wines showed a trend of increasing and then decreasing during the alcoholic fermentation and maturation period. For all the wines, during the alcoholic fermentation, the anthocyanin contents increased significantly along with the increase in alcohol concentration and maceration time, and then decreased rapidly during the maturation period. The peak values of the cyanidin-3-*O*-galactoside, cyanidin-3-*O*-glucoside and cyanidin-3-*O*-arabinoside were observed in the second week of wine maturation. The reason is probably that after the end of wine fermentation, the pressing process may release more anthocyanins adsorbed by the solid skin residue to the wine. In addition to the influence of the berry itself, enzyme treatment and fermentation conditions and the disposition of raw materials will also have a great impact on the anthocyanin content of the wine. In this study, the freezing treatment was a special disposal method. In the process of freezing, water evaporation and berry shrinkage lead to the increase of anthocyanin content in berries. The freezing treatment can largely destroy the cytoarchitecture of berries, promoting the release of anthocyanins in cells. Therefore, the anthocyanin concentration of the wine made from frozen berries was higher than that made from fresh berries [24]. It is noteworthy that the maceration process also significantly affects the anthocyanin content in wine. Carbonic maceration is a special case for red wines. Under an anaerobic environment, intracellular fermentation occurs inside the whole berries, causing the production of alcohol, the degradation of malic acid and the diffusion of phenolic compounds from the skin to the pulp. Intracellular fermentation reduces the extraction rate of anthocyanins in the skin, so the anthocyanin content of red wines made by carbonic maceration is generally lower than that of wines made by traditional methods [25]. However, in this study, carbonic maceration did not seem to significantly reduce the content of anthocyanins in black chokeberry wine. It is hypothesized that the first reason was that the anthocyanin content in the black chokeberries was higher than in grapes, and the second reason was that the anthocyanins in red grapes were presented in the skins, whereas the anthocyanins were abundant in whole black chokeberries, both in the skins and in the pulp.

As a consequence, all four alternative methods, as compared to traditional fermentation, should increase the anthocyanin content in black chokeberry wines to some extent. Frozen fruit fermentation could significantly enhance the content of cyanidin-3-*O*-galactoside, cyanidin-3-*O*-glucoside and cyanidin-3-*O*-arabinoside, and carbonic maceration and co-carbonic maceration could increase the content of cyanidin-3-*O*-galactoside and cyanidin-3-*O*-glucoside in the wine. The co-fermentation was able to increase the content of cyanidin-3-*O*-glucoside in the wine. Overall, the frozen fruit fermentation method appeared to be the most effective in increasing the content of anthocyanins in black chokeberry wine.

##### Variation of Non-Anthocyanic Phenolic Compounds

**Figure 3:**
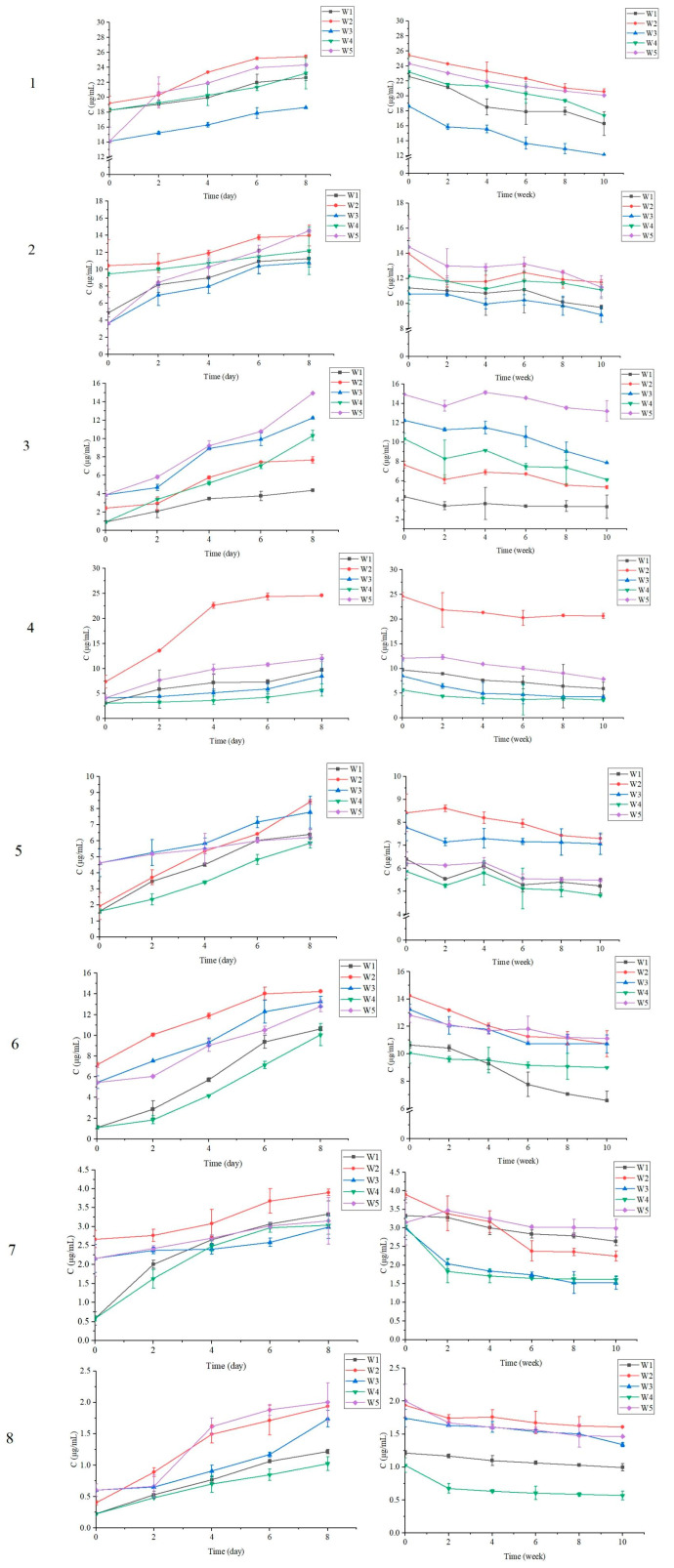
Variation in the contents of non-anthocyanic phenolic compounds in black chokeberry wines during the alcoholic fermentation period (left graph) and maturation period (right graph). (**1**). Chlorogenic acid, (**2**). Epicatechin, (**3**). Caffeic acid, (**4**). Rutin, (**5**). Astragalin, (**6**). Isoquercitrin, (**7**). Myricetin, (**8**). Quercetin. W1—wine made by traditional fermentation; W2—wine made by frozen fruit fermentation; W3—wine made by co-fermentation; W4—wine made by carbonic maceration; W5—wine made by co-carbonic maceration.

The variation of the contents of chlorogenic acid, epicatechin, caffeic acid, rutin, astragalin, isoquercetin, myricetin and quercetin from fermentation to maturation were monitored by HPLC and the results are presented in Figure 3. According to Figure 3, the contents of the eight non-anthocyanic phenolic compounds in all experimental black chokeberry wines increased significantly during the period of alcoholic fermentation and then decreased during the first stage of the maturation period, followed by a trend toward stability.

The content of non-anthocyanic phenolic compounds decreased during the maturation period, due probably to the oxidation and condensation of phenolic substances in the black chokeberry wines. All four alternative brewing methods increased the content of phenolic compounds compared to TF. It is well-reported that vinification by using FF [26], Co-F [27] and CM [28] technologies can enhance the polyphenol contents in red wines. The results obtained by the present work indicate the potential application of these alternative technologies to black chokeberry winemaking from the point of view of increasing phenolic levels in the final products.

Furthermore, by using the FF method, the content of seven non-anthocyanic phenolic compounds, including chlorogenic acid, epicatechin, caffeic acid, rutin, asiaticoside, isoquercitrin and quercetin increased. The Co-F method effectively increased the content of four phenolic components of caffeic acid, astragalin, isoquercitrin and quercetin in the wine. The CM method also significantly increased the contents of four individual polyphenols in black chokeberry wine, namely chlorogenic acid, epicatechin, caffeic acid and isoquercitrin. The Co-CM method was able to increase the contents of eight phenolic components in black chokeberry wine: chlorogenic acid, epicatechin, caffeic acid, rutin, astragalin, isoquercitrin, myricetin and quercetin.

According to the results obtained above, it can be concluded that all proposed alternative brewing technologies could increase not only anthocyanins, but also non-anthocyanic phenolic compounds; particularly frozen fruit fermentation and co-carbonic maceration.

### 3.2. Enological Parameters of Black Chokeberry Wines at the Time of Bottling

The enological parameters of black chokeberry wine produced by the five brewing methods are shown in Table 3. The results show that the four alternative modified methods had significant differences compared with the TF method, especially regarding the total acid, alcohol concentration and pH, but the method did not significantly affect reducing sugar. Compared with the TF method, four alternative methods can effectively improve the pH of black chokeberry wine. Among them, Co-F, CM and Co-CM can reduce the total acid content of black chokeberry wines. Our results indicate that those methods had positive effects on reducing the excessive acidity of traditional black chokeberry wines. The higher alcohol content of Co-F made wine produced by that method taste more full-bodied.

### 3.3. Analysis of Polyphenols and Volatile Compounds in the Black Chokeberry Wines at the Time of Bottling

#### 3.3.1. Analysis of Phenolic Compounds

The phenolic contents in black chokeberry wine produced by five brewing methods are shown in Table 4. The results show that the four alternative modified methods had significant differences compared with the TF method. Compared with TF, the FF, Co-F, CM and Co-CM methods significantly increased the contents of 10, 5, 6 and 9 individual polyphenols in black chokeberry wines, respectively. Among them, FF wine had the best enhancement effect. The content of chlorogenic acid, epicatechin, rutin, astragalin, quercetin, cyanidin-3-*O*-galactoside, cyanidin-3-*O*-glucoside and cyanidin-3-*O*-arabinoside in FF wine was the highest among the five wines. Co-CM increased the contents of 10 phenolic substances in the wine, including chlorogenic acid, epicatechin, caffeic acid, rutin, astragalin, isoquercitrin, myricetin, quercetin, cyanidin-3-*O*-galactoside and cyanidin-3-*O*-glucoside. Co-F increased the content of caffeic acid, astragalin, isoquercitrin, quercetin and cyanidin-3-*O*-glucoside in black chokeberry wine. CM increased the contents of chlorogenic acid, epicatechin, caffeic acid, isoquercitrin, cyanidin-3-*O*-galactoside and cyanidin-3-*O*-glucoside in the wine. The four brewing methods reduced the total flavonoids content in the wine, which were lower than that produced by TF. However, FF and CM increased the content of total polyphenols in black chokeberry wine, and FF also increased the content of total anthocyanins and total proanthocyanidins in the wine. Because polyphenols are well-known bioactive compounds, the proposed alternative technologies may have practical significance in producing polyphenol-rich or health-beneficial wines.

#### 3.3.2. Analysis of Volatile Compounds

##### Qualitative Analysis

From the qualitative analysis (as shown in Table 5), a total of 62 volatile compounds were identified in different black chokeberry wines, including 19 alcohols, 17 esters, 6 aldehydes, 5 phenols, 4 ketones, 4 acids and 7 alkanes. Among them, 8 alcohols, 5 esters, 3 aldehydes, 1 phenol, 3 ketones, 2 acids and 2 hydrocarbons were found in all five black chokeberry wines. The most abundant volatile components were detected in the wine made by FF, with a total of 53 aroma components identified, followed by Co-CM, CM and Co-F, which exhibited 52, 50 and 43 aroma components, respectively; whereas the fewest components, i.e., only 34 volatile compounds, were identified in the wine made by TF.

Compared with TF, FF, Co-CM, CM and Co-F are all recommended to potentially improved the complexity of aroma of black chokeberry wines, to varying degrees. The volatile compounds in the wine made by FF increased by 56% in contrast with those produced in wine by TF. The probable cause of this phenomenon was that the freezing process destroyed the cell structure and lead to the change in berry cell permeability, which was conducive to the extraction of aroma components in the skin. The varieties of volatile compounds in the wine made by CM increased by 47% as compared to those in wine made from the traditional method. The berry undergoes intracellular fermentation under anaerobic conditions. This process could promote the formation of aroma precursors, which can effectively improve the aroma defects of traditional black chokeberry wine. The varieties of volatile compounds in the Co-F and Co-CM wines increased by 26% and 53%, respectively. The results show that co-fermentation could be a potential technology to improve the aroma complexity of fruit wine, and co-carbonic maceration would have more obvious advantages in aroma enhancement.

##### Quantitative Analysis

Aroma is one of the most important factors that influence the quality of fruit wines. The perception of the aroma of fruit wines is the result of various interactions between a large number of chemical components and sensory receptors, and this special interaction determines the final aroma presentation of fruit wine. Table 6 presents the results obtained by the quantitative analysis of volatile aroma compounds in the black chokeberry wines made by five different brewing technologies.

It can be observed in Table 6 that different brewing techniques can significantly affect the contents of volatile aroma compounds. The total concentrations of volatile compounds varied from 1468 mg/L to 2165 mg/L, with the highest concentration for Co-CM wine and FF wine, followed by Co-F wine and TF wine, whereas CM wine presented the lowest concentration of total volatile aroma compounds. These results indicate that the Co-F and FF methods can effectively enhance the aroma intensity of black chokeberry wine; moreover, different brewing technologies also had a significant impact on the types of aroma substances. A total of 29 aroma components were quantified in wine brewed by TF, including 9 alcohols, 8 esters, 3 aldehydes, 1 phenol, 3 ketones, 2 acids and 3 hydrocarbons. A total of 48 aroma components were quantified in wine brewed by FF, including 16 alcohols, 14 esters, 4 aldehydes, 3 phenols, 4 ketones, 3 acids and 4 hydrocarbons. A total of 39 aroma components of wine brewed by Co-F were quantified, including 16 alcohols, 9 esters, 4 aldehydes, 2 phenols, 4 ketones, 2 acids and 2 hydrocarbons. A total of 44 aroma components of wine brewed by CM were quantified, including 18 alcohols, 11 esters, 3 aldehydes, 3 phenols, 4 ketones, 3 acids and 2 hydrocarbons. A total of 47 aroma components of wine brewed by Co-CM were quantified, including 16 alcohols, 15 esters, 3 aldehydes, 3 phenols, 4 ketones, 3 acids and 3 hydrocarbons. Compared with the traditional method, the types of aroma substances in wines made by the four alternative brewing technologies, i.e., FF, Co-F, CM and Co-CM, increased by 65.5%, 34.5%, 51.7% and 62.1%, respectively. On the basis of these results, it can also be predicted that the aroma complexity of the black chokeberry wines made by the four alternative brewing technologies could be significantly increased, thus improving their sensory quality.

Alcohols are also an important part of the aroma composition and contribute greatly to the flavor of the wines, giving the wine body floral and fruity aromas. The alcohol contents in the wine produced by the five methods were between 731.7 mg/L and 1226 mg/L. Except for the Co-CM, the alcohol contents in the wine of the other alternative technologies were lower than those of TF, indicating that Co-CM had the greatest effect on the increase of alcohol in the black chokeberry wine. Furthermore, in black chokeberry wines produced by the five different brewing technologies (TF, FF, Co-F, CM and CO-CM), alcohols account for 74%, 49%, 60%, 50% and 57% of the total aroma substance content, respectively. A total of 19 alcohols were determined in the five wines. In four non-traditional black chokeberry wines, ten alcohols not contained in traditional wines were detected, such as 3-methyl-1-butanol, (*Z*)-3-hexen-1-ol, and 2-heptanol. These substances were able to provide the fragrance of flowers, fruits and spices for the black chokeberry wine. The increase in the diversity and intensity of alcohols might contribute to black chokeberry wine having more complexity and tremendous aroma.

The esters were mainly derived from yeast metabolism during fermentation and from the leaching of aroma-presenting substances from the berries. For the esters, their contents were 233.8 mg/L, 683.3 mg/L, 394.9 mg/L, 549.4 mg/L and 629.9 mg/L in the TF, FF, Co-F, CM and Co-CM, respectively. In other words, the other four alternative brewing technologies significantly increased the contents of esters in the wines compared to TF; especially, FF exhibited the greatest effect on the content of esters in black chokeberry wine. A total of fifteen esters were determined in the five wines. In four non-traditional black chokeberry wines, seven esters not contained in traditional wines were detected, such as octanoic acid, ethyl ester, hexanoic acid, hexyl ester, decanoic acid, and methyl ester. These substances were able to provide the fragrance of fruits, butters and herbs for the black chokeberry wine. Compared with TF, the composition and content of esters in the wines made by alternative brewing technologies were significantly increased, indicating that the FF, Co-F, CM and Co-CM methods were more efficient at extracting and releasing aroma substances from the berries into wines, and thus may provide black chokeberry wines with higher aroma quality.

For aldehydes, their contents in the wines made by different brewing technologies ranged from 26.58 mg/L to 103.0 mg/L. Compared to TF, FF, Co-F and Co-CM showed higher levels of aldehydes. The maximum content was observed in the wines made by Co-CM, indicating that the Co-CM method had the greatest effect on the aldehyde content. A total of four aldehydes were determined in the five wines made by different brewing technologies; these were benzaldehyde, decanal, 2,5-dimethylbenzaldehyde and 2,4-dimethylbenzaldehyde. Benzaldehyde, 2,5-dimethylbenzaldehyde and 2,4-dimethylbenzaldehyde were identified in TF, CM and Co-CM black chokeberry wine. The above three substances were able to provide nutty aromas such as bitter almond to the wine. All four aldehydes were detected in the wines brewed by FF and Co-F. They contributed not only nutty aromas, but also refreshing citrus fruit and floral aromas to the wine.

The results of the volatile phenolics determination showed that the highest level of 7.145 mg/L was found in Co-F, whereas the lowest level was 0.9910 mg/L in TF. Co-F had the greatest effect on the phenolic content of the black chokeberry wine. A total of four volatile phenolics were determined in the five wines made by different brewing technologies, namely butylhydroxytoluene, 4-vinylguaiacol, 2,4-di-tert-butylphenol and 3,4,5-trimethylphenol. These substances gave the wine a smoky and woody aroma.

For ketones, the highest content, of 87.14 mg/L, was found in the Co-F wine, whereas the lowest content was 62.39 mg/L in the TF wine, indicating that the use of Co-F had the greatest effect on the ketone content of black chokeberry wine. A total of four ketones were determined for the five wines, namely, acetoin, 2-nonanone, damascenone and acetovanillone. Three ketones, acetoin, 2-nonanone and acetovanillone, were detected in the TF wine, which mainly provided the fermented aroma of milky, buttery and vanillin flavors for black chokeberry wine. In addition to the above three components, damascenone was also detected in the other four kinds of black chokeberry wine. This substance provides elegant floral and fruity odors of roses, plums and raspberries. The rich aroma varieties increased the diversity and hierarchy of aroma of black chokeberry wine.

From the results of the volatile acid determination, its content in the five wines was between 3.753 mg/L and 6.096 mg/L. Compared to TF, the volatile acid contents in wines made by other methods was higher. The largest increase was observed in the Co-CM wine, indicating that Co-CM had the greatest effect on the acid content. Hexanoic acid and benzoic acid were detected in wines made with TF. These two substances contribute to the odors of “cheesy” and “balsamic”. In addition to the above two substances, octanoic acid was also detected in the FF, CM and Co-CM wines. Octanoic acid provides a weak soapy odor for wine, which may have a negative impact on the black chokeberry wines.

The contents of hydrocarbons in the five wines ranged between 21.62 mg/L and 112.8 mg/L. Although the hydrocarbons in wines of different methods showed significant changes compared to TF, the method with the greatest change in content was FF. A total of four hydrocarbons were determined in the five wines, namely p-xylene, dodecane, styrene and 1,2,4,5-tetramethylbenzene, which gave a slight floral and plastic taste to the black chokeberry wines.

Although aldehydes, ketones and acids accounted for only 7–10% of the total aroma substances, they showed a positive effect on the aromatic diversity and complexity layering of wines. With the exception of TF, all four alternative technologies imparted strong floral and fruity aromas to the wines.

Previous study has reported that different brewing methods would lead to great differences in the type and content of aroma components in fruit wine [28,29]. In the present work, all four alternative technologies could enhance the complexity and richness of wine aroma to a certain extent, among which the FF, Co-F, CM methods can best improve the aroma quality of black chokeberry wines. The latter results are consistent with previous reports on red winemaking [9,14,30].

### 3.4. Sensory Evaluation of Black Chokeberry Wine by Tasting Panel

The different brewing methods affected the sensory quality of black chokeberry wine. As shown in Table 7, sensory evaluation scores of the wines made by the other four brewing methods were higher than those of the traditional method. In terms of wine appearance, the highest score was obtained by the FF, which showed a deep purplish red color, differing significantly from the medium purplish red color of the other methods, wherein the higher anthocyanins content gave the wine a deeper color. In terms of aroma, the Co-CM method scored the highest, with the advantages of both Co-F and CM, resulting in higher aromatic complexity, intensity and hierarchy compared to the other methods. In terms of taste, the Co-F scored higher than the others. The smooth and silky tannins of Cabernet Sauvignon improved the rough taste of the black chokeberries, whereas the blend with black chokeberries reduced its excessive acidity and enhanced the sensory quality of the black chokeberry wine. In terms of finish, there was no significant difference in the scores among the five methods. In terms of overall impression, the wine made with Co-CM was superior to the wine made by the other methods. The wine not only had the typical characteristics of black chokeberry and Cabernet Sauvignon, but also had a medium purple-red appearance, rich fruit and floral aromas, and a well-balanced and harmonious palate, which made it a wine with the layers and complexity expected of a black chokeberry dry red wine, thus earning it a higher overall impression score.

In conclusion, the brewing methods used in this study had positive implications for the improvement of the sensory quality of black chokeberry wine. In terms of improving the wine sensory quality, the Co-CM method was superior to Co-F, which was superior to FF, which was superior to CM.

## 4. Conclusions

This was the first time that some alternative grape winemaking technologies were applied to black chokeberry brewing. Taking the phenolic composition and volatile aroma compounds and sensory quality as the main evaluation indexes, the effects of five different brewing technologies on black chokeberry wines were comprehensively analyzed. Compared with traditional fermentation, the four alternative brewing technologies resulted in lower acidity, softer taste, richer aroma, and higher polyphenol contents, thus improving the overall quality of the black chokeberry wines. Using the four alternative brewing technologies, the contents of several major phenolic compounds in black chokeberry wines were increased and the aroma types were also increased by 34.5–65.5%. The volatile aroma substance contents of wines brewed by frozen fruit fermentation, co-fermentation and co-carbonic maceration were higher than with that of traditional fermentation. The results of a sensory evaluation by a tasting panel were consistent with those obtained by chemical analysis. The black chokeberry wines made by the four alternative technologies have higher sensory evaluation scores than that made by traditional technology, due essentially to their elegant appearance, rich aroma, balanced taste and long aftertaste. The alternative brewing technologies proposed by this work provide important practical applications for producing quality black chokeberry as well as other fruit wines.

## Figures and Tables

**Table 1:** Evaluation of wine sensory qualities.

	Appearance, 3 Max	Aroma, 6 Max	Taste and Texture, 6 Max	Aftertaste, 3 Max	Overall Impression, 2 Max	Total Scores
Grades	3—Excellent -clear and lustrous, with the typical color. 2—Good -Clear with characteristic color. 1—Poor -Slightly hazy and cloudy or slightly lusterless. 0—Objectionable -Cloudy or lightless wine.	6—Extraordinary -Very typical berry or floral aromas, very rich and harmonious. 5—Excellent -Typical fruity or floral aroma, full-bodied and harmonious. 4—Good -Typical fruity or floral aroma, mellow and prominent. 3—Acceptable -Slightly fruity or floral, with a slight mellow aroma. 2—Deficient -No fruity or mellow aroma, or slight odor. 1—Poor -Very poor, smells bad. 0—Objectionable -Bad odor	6—Extraordinary -A very typical wine with a balanced, round, rich and full-bodied taste. 5—Excellent -With the above characteristics but a little less, elegant but not mellow. 4—Good -Typical wine taste, balanced, round and fuller. 3—Acceptable -No typicality but pleasant, slightly lean or rough on the palate. 2—Deficient -No typicality, more disadvantages than above. 1—Poor -Unpleasant taste, poor balance or bad sense of structure. 0—Objectionable -A disgusting taste or structure.	3—Excellent -Lingering, outstanding aftertaste. 2—Good -Longer after-taste 0—Objectionable -No after-taste	2—Excellent 1—Good 0—Poor	18—20 Extraordinary 15—17 Excellent 12—14 Good 9—11 Commercially Acceptable 6—8 Deficient 0—5 Poor and objectionable

**Table 2:** Characterization of anthocyanins in black chokeberry wines.

No.	RT(min)	Compound	Formula	Measured [M+H]^+^/(Da)	Theoretical [M+H]^+^/(Da)	Error (ppm)	Product Ions m/z(Da)	Wine Sample
1	1.92	Cyanidin-3,5-*O*-diglucoside	C_27_H_31_O_16_^+^	611.1611	611.1606	0.8	449.1040/287.0540	W1, W2, W3, W4, W5
2	2.42	Cyanidin-3-*O*-galactoside	C_21_H_21_O_11_^+^	449.1059	449.1078	−4.2	287.0537	W1, W2, W3, W4, W5
3	3.77	Cyanidin-3-*O*–glucoside	C_21_H_21_O_11_^+^	449.1059	449.1078	−4.2	287.0537	W1, W2, W3, W4, W5
4	7.08	Cyanidin-3-*O*-arabinoside	C_20_H_19_O_10_^+^	419.0981	419.0972	2.1	287.0550	W1, W2, W3, W4, W5
5	12.10	Malvidin-3-*O*-glucoside	C_23_H_25_O_12_^+^	493.1357	493.1340	3.4	331.0813	W3, W5
6	12.75	Cyanidin-3-*O*-xyloside	C_20_H_19_O_10_^+^	419.0978	419.0972	1.4	287.0550	W1, W2, W3, W4, W5
7	13.81	Peonidin-3,5-*O*-diglucoside	C_28_H_33_O_16_^+^	625.1713	625.1763	8.0	463.1193/301.0678	W3, W5
8	16.20	Malvidin-3-(6”-caffeoyl)-glucoside	C_32_H_33_O_15_^+^	655.1668	655.1654	2.1	493.1366/331.0782	W3, W5

W1—wine made by traditional fermentation; W2—wine made by frozen fruit fermentation; W3—wine made by co-fermentation; W4—wine made by carbonic maceration; W5—wine made by co-carbonic maceration.

**Table 3:** Enological parameters of black chokeberry wines.

Wine Sample	W1	W2	W3	W4	W5
Alcohol(%vol)	12.30 ± 0.10 b	12.43 ± 0.12 ab	12.68 ± 0.18 a	12.37 ± 0.15 b	12.50 ± 0.20 ab
Total acid(g/L)	8.77 ± 0.04 a	8.65 ± 0.08 ab	8.54 ± 0.04 b	8.30 ± 0.11 c	8.26 ± 0.10 c
Reducing sugar(g/L)	3.82 ± 0.12 a	3.87 ± 0.05 a	3.84 ± 0.08 a	3.73 ± 0.10 a	3.75 ± 0.05 a
pH	3.60 ± 0.02 c	3.72 ± 0.05 b	3.67 ± 0.03 b	3.81 ± 0.04 a	3.85 ± 0.00 a

Values with the different letters (a to c) in the same column indicate significant statistical differences (*p* < 0.05). W1—wine made by traditional fermentation; W2—wine made by frozen fruit fermentation; W3—wine made by co-fermentation; W4—wine made by carbonic maceration; W5—wine made by co-carbonic maceration.

**Table 4:** Phenolic contents in the black chokeberry wines made by different brewing technologies.

Wine Sample	W1	W2	W3	W4	W5
Chlorogenic acid (μg/mL)	16.28 ± 1.60 b	20.56 ± 0.37 a	12.14 ± 0.02 c	17.39 ± 0.03 b	20.09 ± 0.11 a
Epicatechin (μg/mL)	9.71 ± 0.16 b	11.70 ± 0.20 a	9.1 ± 0.61 b	11.08 ± 0.56 a	11.31 ± 0.91 a
Caffeic acid (μg/mL)	3.30 ± 1.21 d	5.32 ± 0.15 c	7.88 ± 0.01 b	6.12 ± 0.08 c	13.21 ± 1.07 a
Rutin (μg/mL)	5.93 ± 1.31 c	20.66 ± 0.52 a	4.22 ± 0.06 d	3.64 ± 0.24 d	7.88 ± 0.03 b
Astragalin (μg/mL)	5.23 ± 0.31 bc	7.29 ± 0.17 a	7.06 ± 0.47 a	4.82 ± 0.04 c	5.49 ± 0.08 b
Isoquercitrin (μg/mL)	6.58 ± 0.66 c	10.72 ± 0.95 a	10.71 ± 0.65 a	9.00 ± 0.02 b	11.12 ± 0.02 a
Myricetin (μg/mL)	2.64 ± 0.12 b	2.24 ± 0.13 c	1.52 ± 0.17 d	1.61 ± 0.10 d	2.99 ± 0.24 a
Quercetin (μg/mL)	0.99 ± 0.06 d	1.61 ± 0.00 a	1.34 ± 0.03 c	0.57 ± 0.06 e	1.46 ± 0.01 b
Cyanidin-3-*O*-galactoside (μg/mL)	168.79 ± 14.96 c	240.77 ± 0.36 a	138.58 ± 0.00 d	194.56 ± 11.3 b	204.81 ± 0.04 b
Cyanidin-3-*O*-glucoside (μg/mL)	12.48 ± 0.14 e	28.00 ± 0.02 a	23.53 ± 1.00 b	22.06 ± 0.15 c	14.79 ± 0.02 d
Cyanidin-3-*O*-arabinoside (μg/mL)	140.34 ± 0.20 b	211.68 ± 23.1 a	98.97 ± 0.01 d	125.93 ± 0.00 bc	110.18 ± 0.00 cd
Total polyphenols (g/L)	7.52 ± 0.08 b	8.13 ± 0.19 a	6.11 ± 0.24 c	7.94 ± 0.09 a	5.95 ± 0.05 c
Total proanthocyanidins (g/L)	1.30 ± 0.00 b	1.44 ± 0.06 a	0.64 ± 0.10 d	0.95 ± 0.01 c	0.71 ± 0.01 d
Total anthocyanins (g/L)	0.74 ± 0.04 b	1.01 ± 0.01 a	0.52 ± 0.05 c	0.59 ± 0.09 c	0.41 ± 0.05 d
Total flavonoids (g/L)	6.52 ± 0.07 a	5.11 ± 0.07 b	3.88 ± 0.06 d	4.74 ± 0.13 c	4.62 ± 0.16 c

Values with the different letters (a to e) in the same column indicate significant statistical differences(*p* < 0.05). W1—wine made by traditional fermentation; W2—wine made by frozen fruit fermentation; W3—wine made by co-fermentation; W4—wine made by carbonic maceration; W5—wine made by co-carbonic maceration.

**Table 5:** Key volatile compounds identified in the black chokeberry wines made by different brewing technologies.

RT	Compounds	RIL	RI	Basis of Identification	Odor	Wine Sample
Alcohols						
6.02	1-Propanol, 2-methyl-	1086	1086	MS RIL	Whiskey	W1, W2, W3, W4, W5
6.20	2-Pentanol	1094	1100	MS RIL	Banana, Apple	W1, W2, W3, W4, W5
10.66	1-Pentanol	1210	1206	MS RIL	Fruity	W1, W2, W3, W4, W5
12.03	1-Butanol, 3-methyl-	1238	1237	MS RIL	Sweet fruity	W2, W3, W5
12.73	2-Heptanol	1252	1286	MS RIL	Lemon grass, Sweet floral	W2, W3, W4, W5
17.72	1-Propanol, 3-ethoxy-	1347	1359	MS RIL	Fruity	W1, W2, W4
18.21	1-Hexanol	1356	1356	MS RIL	Green fruity, Apple	W1, W2, W3, W4, W5
19.78	3-Hexen-1-ol, (*Z*)-	1384	1337	MS RIL	Grass, Herbal	W2, W3, W4, W5
28.23	1-Hexanol, 2-ethyl-	1530	1522	MS RIL	Citrus, Floral	W1, W2, W3, W4, W5
28.82	2,3-Butanediol	1540	1539	MS RIL	Creamy, Buttery	W1, W2, W3, W4, W5
29.97	1-Octanol	1561	1561	MS RIL	Citrus	W2, W3, W4, W5
31.80	Terpinen-4-ol	1592	1593	MS RIL	Peppery, Woody	W2, W3, W4, W5
37.40	3,6-Nonadien-1-ol, (*E,Z*)-	1690	1731	MS RIL	Cucumber, Melon	W2, W3, W4, W5
46.41	Benzyl Alcohol	1855	1855	MS RIL	Cherry, Almond	W1, W2, W3, W4, W5
48.10	Phenylethyl Alcohol	1886	1888	MS RIL	Rose, Honey	W1, W2, W3, W4, W5
51.49	1,5-Pentanediol, 3-methyl-	1938	-	MS	-	W2, W3, W4, W5
54.89	3-Phenylpropanol	2010	2022	MS RIL	Cinnamon, Fruity	W4, W5
63.61	1- Dodecanol	2177	1983	MS RIL	Honey, Coconut	W3, W4
64.42	Tetradecanol	2190	2175	MS RIL	Fruity	W4
Esters						
4.79	Butanoic acid, ethyl ester	1036	1040	MS RIL	Pineapple, Strawberry	W3, W5
7.11	1-Butanol, 3-methyl-, acetate	1120	1121	MS RIL	Banana, Pear	W1, W2, W5
11.66	Hexanoic acid, ethyl ester	1230	1230	MS RIL	Orris, Herbal, Carrot	W1, W2, W3, W4, W5
13.01	2-Hexenoic acid, ethyl ester	1258	1329	MS RIL	Grape, Rum	W1, W2, W4, W5
17.29	Propanoic acid, 2-hydroxy-, ethyl ester	1330	1331	MS RIL	Pineapple, Buttery	W1, W2, W3, W4, W5
22.51	Octanoic acid, hexyl ester	1431	1431	MS RIL	Apple	W2, W3, W4, W5
33.10	Hexanoic acid, hexyl ester	1591	1596	MS RIL	Herbaceous, Fruity, Vegetable	W2, W3, W4, W5
34.14	Nonanoic acid, ethyl ester	1607	1581	MS RIL	Fruity, Rose, Waxy, Rum	W1, W2, W3, W4, W5
34.87	Benzoic acid, ethyl ester	1646	1647	MS RIL	Cherry, Mint	W1, W2, W3, W4, W5
36.18	Butanedioic acid, diethyl ester	1669	1668	MS RIL	Apple, Apricot, Cranberry	W1, W2, W3, W4, W5
36.26	Decanoic acid, methyl ester	1672	1636	MS RIL	Wine, Fruity	W2, W3, W5
38.45	Acetic acid, phenylmethyl ester	1709	1710	MS RIL	Jasmine	W4
41.58	Benzeneacetic acid, ethyl ester	1760	1760	MS RIL	Honey, Rose, Cocoa	W1, W2, W3, W5
53.20	D-octanolactone	1990	1999	MS RIL	Coconut, Creamy	W2, W4, W5
54.34	Pantolactone	2009	2006	MS RIL	Cotton Candy	W2, W4, W5
58.10	Butanedioic acid, hydroxy-, diethyl ester	2059	2060	MS RIL	Fruity, Herbal	W5
58.27	Ethyl (*Z*)-cinnamate	2082	2081	MS RIL	-	W2, W4, W5
Aldehydes						
20.62	Nonanal	1405	1405	MS RIL	Citrus	W2, W4
26.61	Benzaldehyde	1502	1502	MS RIL	Almond, Nutty	W1, W2, W3, W4, W5
27.50	Decanal	1514	1515	MS RIL	Citrus, Floral	W2, W3
33.39	Benzeneacetaldehyde	1622	1622	MS RIL	Grapefruit, Honey, Hyacinth, Lemon	W1
38.28	Benzaldehyde, 2,5-dimethyl-	1706	1705	MS RIL	-	W1, W2, W3, W4, W5
38.45	Benzaldehyde, 2,4-dimethyl-	1709	1710	MS RIL	Cherry, Almond, Spice, Vanilla	W1, W2, W3, W4, W5
Phenols						
48.44	Butylated Hydroxytoluene	1892	1902	MS RIL	Camphor	W2, W3, W4, W5
52.85	Phenol	1990	1989	MS RIL	Plastic, Rubber	W1, W2, W3, W4, W5
53.89	Phenol, 4-ethyl-2-methoxy-	1999	2008	MS RIL	Spicy, Smoky	W1
61.71	2-Methoxy-4-vinylphenol	2134	2156	MS RIL	Peppery, Smoky, Woody	W3
68.81	2,4-Di-tert-butylphenol	2249	2277	MS RIL	-	W2, W4, W5
82.30	Phenol, 3,4,5-trimethyl-	2461	-	MS	-	W1, W2, W4, W5
Ketones						
15.05	Acetoin	1299	1302	MS RIL	Milky	W1, W2, W3, W4, W5
19.23	2-Nonanone	1374	1374	MS RIL	Cheesy, Buttery	W1, W2, W3, W4, W5
40.18	Damascenone	1741	1787	MS RIL	Rose, Plum, Raspberry	W2, W3, W4, W5
79.55	Acetovanillone	2419	-	MS	Vanillin	W1, W2, W3, W4, W5
Acids						
33.90	Butanoic acid	1631	1631	MS RIL	Cheesy, Dairy	W1, W2, W3, W4, W5
45.63	Hexanoic acid	1841	1841	MS RIL	Cheesy	W1, W2, W3, W4, W5
56.38	Octanoic acid	2077	2076	MS RIL	Soapy	W2, W3, W4, W5
76.94	Benzoic acid	2381	2387	MS RIL	Balsamic	W1, W2, W4, W5
Hydrocarbons						
7.57	p-Xylene	1132	1132	MS RIL	-	W2, W4, W5
10.16	Dodecane	1200	1200	MS RIL	-	W1, W2, W3
12.45	Styrene	1248	1248	MS RIL	Floral, Plastic	W1, W2, W3, W4, W5
21.70	Benzene, 1,2,4,5-Tetramethyl-	1395	1400	MS RIL	-	W1, W2, W5
32.12	n-Hexadecane	1600	1600	MS RIL	-	W1, W2, W3, W4, W5
33.52	Pentadecane, 2,6,10,14-Tetramethyl-	1621	1655	MS RIL	-	W2, W4, W5

W1—wine made by traditional fermentation; W2—wine made by frozen fruit fermentation; W3—wine made by co-fermentation; W4—wine made by carbonic maceration; W5—wine made by co-carbonic maceration. RI = retention index; RIL = compounds were identified by RI from the literature; MS = compounds were identified by MS spectra. ※RIL are obtained from https://webbook.nist.gov/chemistry/ (accessed on 10 January 2022). Odors are obtained from http://www.thegoodscentscompany.com/index.html (accessed on 13 April 2022).

**Table 6:** Contents of the major volatile compounds in black chokeberry wines made by different brewing technologies (mg/L).

Wine Sample	W1	W2	W3	W4	W5
Alcohols					
1-Propanol, 2-methyl-	17.05 ± 0.66 a	14.37 ± 0.88 c	16.03 ± 1.05 ab	16.41 ± 0.47 ab	15.52 ± 0.54 bc
2-Pentanol	8.730 ± 0.61 a	3.110 ± 0.19 d	4.680 ± 0.12 b	2.360 ± 0.02 e	3.650 ± 0.10 c
1-Pentanol	409.8 ± 7.82 c	367.3 ± 2.65 d	485.3 ± 2.38 b	369.9 ± 1.81 d	618.3 ± 15.78 a
1-Butanol, 3-methyl-	-	2.148 ± 0.05 c	2.508 ± 0.09 b	-	3.495 ± 0.10 a
2-Heptanol	-	60.69 ± 0.56 a	11.15 ± 0.48 b	6.540 ± 0.05 c	10.79 ± 0.12 b
1-Propanol, 3-ethoxy-	11.62 ± 0.33 a	2.599 ± 0.35 c	-	3.537 ± 0.25 b	-
1-Hexanol	25.13 ± 0.28 c	26.41 ± 0.48 b	12.36 ± 0.11 d	31.16 ± 0.70 a	25.84 ± 0.12 bc
3-Hexen-1-ol, (*Z*)-	-	13.10 ± 0.19 d	16.92 ± 0.21 c	48.60 ± 0.84 b	56.54 ± 0.68 a
1-Hexanol, 2-ethyl-	83.83 ± 1.92 d	133.5 ± 1.66 a	102.7 ± 1.34 c	59.12 ± 1.55 e	118.8 ± 2.59 b
2,3-Butanediol	18.38 ± 0.36 a	0.7521 ± 0.31 c	1.736 ± 0.00 b	0.1482 ± 0.01 d	2.111 ± 0.24 b
1-Octanol	-	0.7051 ± 0.02 c	1.881 ± 0.06 b	1.818 ± 0.01 b	3.834 ± 0.13 a
Terpinen-4-ol	-	28.77 ± 0.79 a	20.86 ± 0.78 c	7.760 ± 0.10 d	23.12 ± 2.09 b
3,6-Nonadien-1-ol, (*E,Z*)-	-	20.06 ± 0.25 c	25.97 ± 0.26 b	15.53 ± 0.24 d	27.04 ± 0.71 a
Benzyl Alcohol	242.2 ± 3.96 a	3.953 ± 0.30 b	5.518 ± 0.03 b	4.098 ± 0.23 b	7.159 ± 0.42 b
Phenylethyl Alcohol	333.4 ± 0.27 a	167.3 ± 1.55 d	276.3 ± 0.70 c	157.4 ± 3.26 e	304.1 ± 4.94 b
1,5-Pentanediol, 3-methyl-	-	2.354 ± 0.09 c	2.756 ± 0.12 b	3.748 ± 0.20 a	2.951 ± 0.12 b
3-Phenylpropanol	-	-	-	2.669 ± 0.10 b	3.045 ± 0.06 a
1-Dodecanol	-	-	2.897 ± 0.09 a	0.07631 ± 0.00 b	-
Tetradecanol	-	-	-	0.8530 ± 0.02 a	-
Total alcohols	1150	847.1	989.6	731.7	1226
Esters					
1-Butanol, 3-methyl-, -acetate	34.05 ± 0.28 a	22.23 ± 0.01 c	-	-	31.62 ± 0.20 b
Hexanoic acid, ethyl ester	29.76 ± 0.06 a	26.82 ± 0.05 c	25.45 ± 0.06 d	25.71 ± 0.39 d	28.43 ± 0.00 b
2-Hexenoic acid, ethyl ester	35.61 ± 1.36 b	38.38 ± 0.32 a	-	29.31 ± 0.17 d	33.77 ± 0.24 c
Propanoic acid, 2-hydroxy-, ethyl ester	32.57 ± 0.54 d	38.78 ± 0.26 b	46.59 ± 0.42 a	36.93 ± 0.68 c	46.95 ± 0.06 a
Octanoic acid, ethyl ester	-	120.9 ± 0.76 c	135.1 ± 0.19 b	91.93 ± 1.32 d	148.4 ± 0.53 a
Hexanoic acid, hexyl ester	-	29.02 ± 0.22 b	31.76 ± 0.56 a	28.59 ± 0.30 b	31.89 ± 0.36 a
Nonanoic acid, ethyl ester	9.497 ± 0.11 c	7.080 ± 0.07 d	7.204 ± 0.01 d	10.94 ± 0.18 b	14.75 ± 0.22 a
Benzoic acid, ethyl ester	46.45 ± 1.83 e	72.76 ± 0.21 c	84.33 ± 1.61 b	63.84 ± 0.06 d	92.93 ± 0.02 a
Butanedioic acid, diethyl ester	7.450 ± 1.13 bc	6.236 ± 0.06 d	6.990 ± 0.39 cd	9.660 ± 0.27 a	8.299 ± 0.11 b
Decanoic acid, methyl ester	-	26.69 ± 0.04 c	30.78 ± 0.20 b	-	42.38 ± 0.44 a
Benzeneacetic acid, ethyl ester	38.41 ± 0.01 a	27.01 ± 0.14 b	26.70 ± 0.28 b	-	26.88 ± 0.09 b
D-octanolactone	-	27.60 ± 0.02 c	-	34.21 ± 0.18 a	31.79 ± 0.05 b
Pantolactone	-	217.8 ± 0.03 a	-	194.2 ± 0.57 b	31.83 ± 0.28 c
Butanedioic acid, hydroxy-, diethyl ester	-	-	-	-	28.26 ± 0.15 a
Ethyl (*Z*)-cinnamate	-	22.05 ± 0.03 c	-	24.15 ± 0.07 b	31.77 ± 0.21 a
Total esters	233.8	683.3	394.9	549.4	629.9
Aldehydes					
Benzaldehyde	13.77 ± 0.76 d	24.17 ± 0.90 b	31.20 ± 0.99 a	18.60 ± 0.03 c	30.89 ± 1.09 a
Decanal	-	8.034 ± 0.04 a	6.534 ± 0.16 b	-	-
Benzaldehyde, 2,5-dimethyl-	11.63 ± 0.77 b	5.510 ± 0.11 d	8.645 ± 0.04 c	0.6581 ± 0.00 e	15.85 ± 0.63 a
Benzaldehyde, 2,4-dimethyl-	14.07 ± 0.15 d	53.79 ± 1.31 b	25.87 ± 0.85 c	7.330 ± 0.28 e	56.26 ± 0.30 a
Total aldehydes	39.47	91.50	72.25	26.58	103.0
Phenols					
Butylated Hydroxytoluene	-	0.7891 ± 0.19 a	0.5392 ± 0.04 ab	0.3483 ± 0.20 b	0.6632 ± 0.07 a
2-Methoxy-4-vinylphenol	-	-	6.606 ± 0.23 a	-	-
2,4-Di-tert-butylphenol	-	0.8012 ± 0.06 a	-	0.5776 ± 0.19 a	0.4791 ± 0.19 a
Phenol, 3,4,5-trimethyl-	0.9910 ± 0.51 a	0.8700 ±0.16 a	-	0.8892 ± 0.34 a	0.6473 ± 0.12 a
Total phenols	0.9910	2.462	7.145	1.814	1.789
Ketones					
Acetoin	29.52 ± 1.96 c	10.79 ± 0.05 d	40.12 ± 2.99 b	33.00 ± 1.85 c	44.05 ± 1.50 a
2-Nonanone	14.89 ± 0.52 a	11.51 ± 0.47 b	11.22 ± 0.34 b	11.85 ± 0.41 b	11.80 ± 0.01 b
Damascenone	-	12.59 ± 0.62 a	12.61 ± 0.18 a	12.35 ± 0.47 a	12.70 ± 0.42 a
Acetoguaiacone	17.98 ± 0.73 d	33.10 ± 0.76 a	23.20 ± 0.11 c	26.65 ± 0.12 b	16.77 ± 1.74 d
Total ketones	62.39	67.99	87.14	83.85	85.32
Acids					
Hexanoic acid	1.716 ± 0.62 c	2.563 ± 0.10 ab	2.838 ± 0.08 a	2.179 ± 0.14 bc	2.998 ± 0.03 a
Octanoic acid	-	0.9571 ± 0.00 b	1.004 ± 0.04 ab	0.8552 ± 0.05 c	1.061 ± 0.02 a
Benzoic acid	2.037 ± 0.06 a	2.012 ± 0.04 a	-	1.753 ± 0.10 b	2.037 ± 0.17 a
Total acids	3.753	5.532	3.842	4.787	6.096
Hydrocarbons					
*p*-Xylene	-	0.3731 ± 0.00 c	-	18.72 ± 1.90 b	22.83 ± 1.32 a
Dodecane	10.43 ± 0.31 c	20.15 ± 0.27 b	21.14 ± 0.46 a	-	-
Styrene	45.14 ± 1.72 d	0.5343 ± 0.00 e	62.87 ± 0.41 b	51.10 ± 1.03 c	69.78 ± 2.46 a
Benzene, 1,2,4,5-tetramethyl-	17.41 ± 0.46 b	0.5661 ± 0.02 c	-	-	20.15 ± 0.23 a
Total hydrocarbons	72.97	21.62	84.01	69.82	112.8
Total	1563	1720	1639	1468	2165

Values with the different letters (a to e) in the same row indicate significant statistical differences (*p* < 0.05). “-” means that the substance was not detected. W1—wine made by traditional fermentation; W2—wine made by frozen fruit fermentation; W3—wine made by co-fermentation; W4—wine made by carbonic maceration; W5—wine made by co-carbonic maceration.

**Table 7:** Scores of sensory characteristics of black chokeberry wines by tasting panel.

Sample	Appearance	Aroma	Taste	Aftertaste	Overall Impression	Total Scores
	3	6	6	3	2	20
W1	2.3	3.9	3.8	1.8	1.3	13.1
W2	2.9	4.5	4.3	1.8	1.1	14.6
W3	2.7	4.6	4.7	1.9	1.2	15.1
W4	2.6	4.1	4.2	2.0	1.3	14.2
W5	2.5	5.4	4.2	1.9	1.6	15.6

W1—wine made by traditional fermentation; W2—wine made by frozen fruit fermentation; W3—wine made by co-fermentation; W4—wine made by carbonic maceration; W5—wine made by co-carbonic maceration.

## Data Availability

The data are available from the corresponding author.
